# Processing ‘Ataulfo’ Mango into Juice Preserves the Bioavailability and Antioxidant Capacity of Its Phenolic Compounds

**DOI:** 10.3390/nu9101082

**Published:** 2017-09-29

**Authors:** Ana Elena Quirós-Sauceda, C.-Y. Oliver Chen, Jeffrey B. Blumberg, Humberto Astiazaran-Garcia, Abraham Wall-Medrano, Gustavo A. González-Aguilar

**Affiliations:** 1 Centro de Investigación en Alimentación y Desarrollo, AC (CIAD, AC), Carretera a la Victoria Km 0.6. La Victoria, Sonora, Hermosillo 83304, Mexico; quirosanaelena@hotmail.com (A.E.Q.-S.); hastiazaran@ciad.mx (H.A.-G.); 2 Antioxidants Research Laboratory, Jean Mayer USDA Human Nutrition Research Center on Aging, Tufts University, 711 Washington Street, Boston, MA 02111, USA; oliver.chen@tufts.edu (C.-Y.O.C.); jeffrey.blumberg@tufts.edu (J.B.B.); 3 Departamento de Ciencias de la Salud, Instituto de Ciencias Biomédicas, Universidad Autónoma de Ciudad Juárez, Anillo Envolvente del PRONAF y Estocolmo s/n, Ciudad Juárez, Chihuahua 32315, Mexico; awall@uacj.mx

**Keywords:** mango, pharmacokinetics, phenolic acids, human, food matrix, antioxidant

## Abstract

The health-promoting effects of phenolic compounds depend on their bioaccessibility from the food matrix and their consequent bioavailability. We carried out a randomized crossover pilot clinical trial to evaluate the matrix effect (raw flesh and juice) of ‘Ataulfo’ mango on the bioavailability of its phenolic compounds. Twelve healthy male subjects consumed a dose of mango flesh or juice. Blood was collected for six hours after consumption, and urine for 24 h. Plasma and urine phenolics were analyzed by electrochemical detection coupled to high performance liquid chromatography (HPLC-ECD). Five compounds were identified and quantified in plasma. Six phenolic compounds, plus a microbial metabolite (pyrogallol) were quantified in urine, suggesting colonic metabolism. The maximum plasma concentration (C_max_) occurred 2–4 h after consumption; excretion rates were maximum at 8–24 h. Mango flesh contributed to greater protocatechuic acid absorption (49%), mango juice contributed to higher chlorogenic acid absorption (62%). Our data suggests that the bioavailability and antioxidant capacity of mango phenolics is preserved, and may be increased when the flesh is processed into juice.

## 1. Introduction

Mango (*Mangifera indica* L.) is one of the most consumed tropical fruits in the world, due to its sensorial attractiveness and nutritional and phytochemical composition [1,2]. Mexico is the leading mango-exporting country (41%), and among Mexican cultivars, ‘Ataulfo’ is one of the most consumed, due to its appetizing organoleptic and sensory characteristics [3]. ‘Ataulfo’ mango has the highest content of phenolic compounds and antioxidant capacity, as compared to other cultivars [2,4]. Because of this, ‘Ataulfo’ mango can be considered a “natural functional food”, whose regular consumption can prevent several chronic diseases, including cardiovascular diseases and some types of cancer [5,6].

To corroborate the health benefits of a functional food, knowledge of its bioactive constituents and their bioaccessibility and bioavailability is required. According to Grundy et al. [7] “Bioaccessibility refers to the proportion of a nutrient or any other substance (i.e., phytochemicals) that is released from the food matrix and is potentially available for absorption in the small intestine”, while bioavailability is defined as the rate and extent to which a compound is absorbed, transported, and becomes available at the site of action [8]. Bioaccessibility precedes bioavailability in nutrient/drug pharmacokinetics and plays a critical role in the final bioefficacy at target organs. The concept of food matrix refers to the fact that nutrients are contained in a continuous medium, where they may interact physicochemically at different length scales with other components and structures, such as proteins, carbohydrates, dietary fiber, lipids, organic acids, and others [9], which governs both the bioaccessibility and bioavailability of nutrients and xenobiotics. Phenolic compounds are generally bound tightly to food matrices [10], consequently, most of the ingested phenolics (67–99%) are not absorbed in the upper gastrointestinal (GI) tract because they are not bioaccessible [11].

The impact of the food matrix on a compound′s bioaccessibility can be strongly influenced by food preparation technologies, such as homogenization, pressing, grinding, fermentation, and heating [9,12,13,14], as well as many intrinsic factors related to the digestive process. Juices are produced by pressing and squeezing the fruit, which releases soluble compounds and those that are not bound to the matrix, resulting in products that differ in nutrient density, sugar profile, pectin, and dietary fiber contents [15]. Phenolic compounds from fruit juices are expected to be more bioaccessible and bioavailable than those from fruit flesh because of their differences in proximate fiber content [5]. Dietary fiber content in ‘Ataulfo’ mango flesh and juice is approximately 18% and 14%, respectively. However, in vivo evidence on the bioavailability of mango phenolics from different matrices has not been fully documented. This information is important to substantiate the reported health benefits of mango phenolics. The objective of this study was to compare the effects of food matrix (flesh and juice) on the bioavailability and antioxidant capacity of phenolic compounds from ‘Ataulfo’ mangos in healthy human subjects.

## 2. Materials and Methods

### 2.1. Chemicals and Solvents

All solvents, salts, and acids used (methanol, HCl, ascorbic acid, ethylenediaminetetraacetic acid (EDTA,) NaH_2_PO_4_, 2′,3′,4′-trihydroxyacetophenone, NaCl, and perchloric acid) were purchased from Fisher Scientific Co (Montreal, Canada). Phenolic standards (*p*-coumaric acid, gallic acid, chlorogenic acid, ferulic acid, vanillic acid, protocatechuic acid, gentisic acid, sinapic acid, caffeic acid, and pyrogallol), β-glucuronidase (containing sulfatase from *Helix pomatia*), and creatinine assay kit were obtained from Sigma-Aldrich (St. Louis, MO, USA).

### 2.2. ‘Ataulfo’ Mangos and Products

Three hundred (200–300 g) commercially ripe (Stage 4) ‘Ataulfo’ mangos [16] were obtained from the local market in Leon, Guanajuato, Mexico and transported to the laboratory. They were washed under tap water, sanitized, peeled, and processed to obtain fresh raw slices (flesh), which were administered to the participants during the crossover intervention. Mango juice was prepared from mango flesh as suggested by Santhirasegaram et al. [17], with some modifications. The flesh was blended using a domestic juice extractor (Moulinex, Centri III, A75312V, Groupe SEB, Mexico City, Mexico) and filtered through organza cloth. The juice was prepared fresh, and immediately consumed by the volunteers.

### 2.3. Phenolic Profile of ‘Ataulfo’ Mango Flesh and Juice

The phenolic composition of the flesh and juice was determined from freeze-dried samples (Labconco Corporation, Kansas City, MO, USA). One gram of the dried products was homogenized in 20 mL of 80% methanol using an IKA^®^ Works homogenizer (Model T25, Willmington, NC, USA) at room temperature. The homogenate was sonicated for 30 min (Bransonic Ultrasonic Co., Model 2210, Danbury, CT, USA) and centrifuged at 20,000× *g* for 15 min at 4 °C. The pellet was homogenized in 10 mL of methanol:water (80/20, *v*/*v*) and reextracted under the same conditions. The supernatants were combined, filtered (Whatman No. 1, Springfield Mill, Maidstone Kent, UK), and made up to a final volume of 30 mL with 80% methanol, to generate a phenolic extract. Before its chromatographic analysis, the extract was subjected to an acid hydrolysis, by adding 1 mL of 2.4 M HCl to 1 mL of the phenolic extract, and incubating the mixture for 2 h in the dark at 80 °C. After the incubation period, the mixture was filtered through a 0.22-µm Ultrafree Durapore Centrifugal filter (Merck Millipore, Billerica, MA, USA), and directly injected into a high pressure liquid chromatography (HPLC) system, equipped with an electrochemical detector (HPLC-ECD) for analysis of phenolic compounds. The HPLC analysis was performed as previously described [18].

### 2.4. Subjects and Study Design

**Figure 1:**
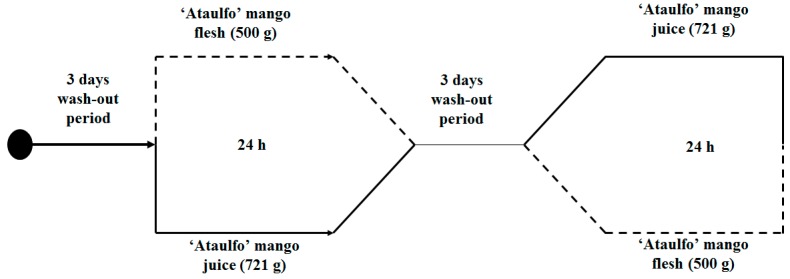
Graphical representation of the randomized crossover clinical trial.

A randomized crossover pilot clinical trial was conducted to evaluate the postprandial plasma bioavailability (0–6 h) and urinary excretion (0–24 h) of phenolic compounds from mango flesh and juice. The protocol was approved by the Bioethics Committee (CE/003/2014) of the Research Center for Food and Development, A.C (CIAD), and was conducted in compliance with the declaration of Helsinki. A graphical representation of the experimental protocol is illustrated in Figure 1. Twelve middle-aged healthy men aged 22 to 34 years were recruited from CIAD. Only male participants were selected to study the bioavailability of mango phenolics, in order to avoid anthropometric variables and menstrual cycle phase-related variability in women that may have affected the absorption, metabolism, and excretion of the compounds of interest. The study was thoroughly explained to the volunteers, and written informed consent was obtained before any experimental activities were performed. All subjects were free of diagnosed heart disease, homeostatic disorders, gastrointestinal disease, or other medical conditions; they were not taking any medication or any vitamin/mineral supplement.

The subjects underwent a 3 day wash-out period to minimize the confounding effect of phenolic compounds from their habitual diets. They were instructed to refrain from consuming foods high in phenolic compounds, such as fruits, berries, vegetables, juices, nuts, tea, coffee, cocoa, olive oil, coffee, wine, and beer. Twenty-four-hour dietary recall was performed, using a slightly modified food frequency questionnaire, to evaluate their compliance to the low phenolic protocol during the wash-out period [19]. At the end of the wash-out period, subjects reported to the study site after a 12 h fast.

After baseline (time 0), blood and urine were collected, subjects were randomly assigned to consume mango flesh slices or juice in a 10-min period, in random order separated by a 3 day wash-out period. The amounts of flesh or juice administered to the subjects (500 g of flesh, 721 g of juice) were chosen because they contained equal amounts of phenolic compounds (45 mg), as determined through chromatographic analyses (see Results). Six additional blood samples were collected at hourly intervals post-consumption, and four additional urine samples were collected at 0–4, 4–8, 8–12, and 12–24 h. Blood was collected in a vacutainer tube containing EDTA as anticoagulant. Plasma was recovered, and 0.5 mL aliquots were immediately acidified using 20 µL of 4 M HCl. Acidified plasma and urine samples were stored at −80 °C until analysis. During blood collection, subjects were only allowed water, other foods and beverages were not allowed. They were given a hamburger without vegetables and ketchup for lunch. For dinner, they were allowed to eat a meal, but with the same restrictions as those of the wash-out period.

### 2.5. Determination of Plasma Phenolic Compounds

Phenolics in plasma were quantified according to the method of Chen, Milbury, Lapsley, and Blumberg [18]. Briefly, 20 µL of vitamin C-EDTA (100 mg ascorbic acid plus 1 mg EDTA in 1.0 mL of 0.4 M NaH_2_PO_4_, pH 3.6), 20 µL of 10 µg/mL internal standard (2′,3′,4′-trihydroxyacetophenone), and 20 µL β-glucuronidase/sulfatase (98,000 kU/L β-glucuronidase and 2400 kU/L sulfatase) were added to 500 µL of plasma, and the mixture was incubated at 37 °C for 60 min. Subsequently, phenolics were extracted with acetonitrile. After centrifugation, the supernatant was removed, dried with nitrogen gas, and reconstituted in the aqueous HPLC mobile phase, filtered using Ultrafree Durapore Centrifugal filter devices (0.22, Merck Millipore, Billerica, MA, USA), and injected into the HPLC- ECD system, using previously optimized chromatographic conditions [18]. The mobile phases were 75 mM citric acid and 25 mM ammonium acetate in 90% water/10% acetonitrile (A), and 75 mM citric acid and 25 mM ammonium acetate in 50% water/50% acetonitrile (B). The concentrations of individual phenolics were calculated based on calibration curves that were constructed using authentic phenolic standards (*R*^2^ > 0.999) of *p*-coumaric acid, gallic acid, chlorogenic acid, ferulic acid, vanillic acid, protocatechuic acid, gentisic acid, sinapic acid, and caffeic acid. The intraday coefficient of variation (CV) for standards spiked into plasma was <8%.

### 2.6. Determination of Urinary Phenolic Compounds

Phenolics in urine were quantified according to the method of McKay et al. [20]. Urine (200 µL) was dissolved in 800 µL of buffer (0.1 M sodium acetate, pH 5) and 30 µL of β-glucuronidase/sulfatase (98,000 kU/L β-glucuronidase and 2400 kU/L sulfatase), and incubated at 37 °C for 60 min. After incubation, 10 µL of 100 µg/mL internal standard (2′,3′,4′-trihydroxyacetophenone) and 200 µL glacial acetic acid were added, and the mixture was briefly vortexed. After the addition of approximately 1 g of NaCl, phenolics were extracted twice with 3 mL of ethyl acetate. The combined ethyl acetate fractions were dried under nitrogen gas and reconstituted in 1 mL of the aqueous HPLC mobile phase, filtered through a 0.22-µm Millex syringe-driven filter (Millipore Corporation, Bedford, MA, USA), and injected (50 µL) onto the HPLC-ECD system. Concentrations of individual phenolics were calculated based on calibration curves constructed using authentic phenolics (*R*^2^ > 0.999), with the adjustment of the internal standard. The intraday CV for standards spiked into urine was <7%. Urinary phenolics were normalized to creatinine concentration, which was measured with a commercial kit.

### 2.7. Antioxidant Capacity

The oxygen radical absorbance capacity (ORAC) assay was performed, according to the method of Prior et al., 2003 [21], using a FLUOstar Optima Microplate reader (BMG Labtechnologies, Inc., Durham, NC, USA). Frozen plasma samples were thawed at room temperature and briefly vortexed. Plasma samples were then mixed with 0.5 M perchloric acid (1:1 *v*/*v*) to obtain a protein-free supernatant. The protein-free supernatants were used in the ORAC assay. Urine samples were analyzed without previous dilution [22].

The ferric reducing antioxidant power (FRAP) assay was performed according to Benzie and Strain [23], using untreated plasma and urine samples. All results are expressed as µmol Trolox equivalents (TE)/mL.

### 2.8. Statistics

Results are expressed as mean ± standard deviation (SD). The maximum concentration (C_max_) in plasma and the time to reach C_max_ (T_max_) were visually identified. The area under the concentration-time curve (AUC) of plasma phenolics was calculated using the linear trapezoidal integration equation [24]. The differences in C_max_, T_max_, and AUC of phenolics between mango flesh and juice treatments were examined using an analysis of covariance, with time 0 values as the covariant, followed by the Tukey-Kramer multi-comparison test. The Number Cruncher Statistical System version 6.0 software (NCSS, NCSS LLC, Kaysville, UT, USA) was used to perform all statistical analyses.

## 3. Results

### 3.1. Mango Phenolics

**Figure 2:**
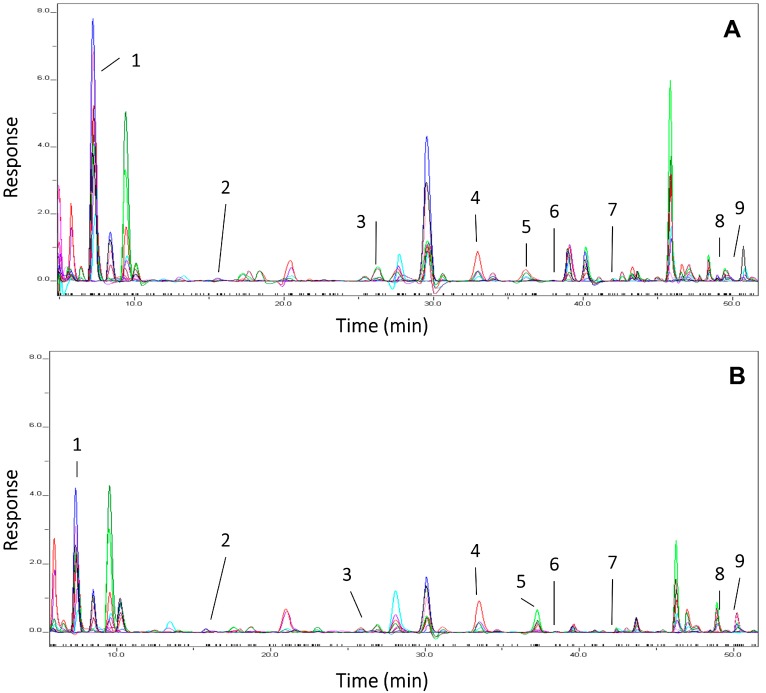
Representative chromatogram obtained by electrochemical detection coupled to high performance liquid chromatography (HPLC-ECD) used to identify and quantify phenolic compounds in mango flesh (**A**) and mango juice (**B**). 1: gallic acid, 2: protocatechuic acid, 3: gentisic acid, 4: chlorogenic acid, 5: vanillic acid, 6: caffeic acid, 7: *p*-coumaric acid, 8: ferulic acid, 9: sinapic acid.

The major phenolics identified in ‘Ataulfo’ mango flesh and juice were gallic, chlorogenic, *p*-coumaric, vanillic, sinapic, protocatechuic, ferulic, gentisic, and caffeic acids (Figure 2 and Table 1). Among the quantified phenolics, *p*-coumaric, gallic, and chlorogenic acids were the predominant species in both flesh and juice. Chlorogenic acid content in juice was 59% lower than in flesh. The sum of quantified phenolics in flesh and juice was 47.60 mg/500 g fresh weight (FW) and 43.24 mg/721 g FW, and were statistically similar. The values reported in Table 1 are the doses effectively consumed by the volunteers (500 g of flesh and 721 g of juice), which contain equal concentrations of phenolic compounds.

### 3.2. Plasma and Urine Pharmacokinetic Analyses

**Figure 3:**
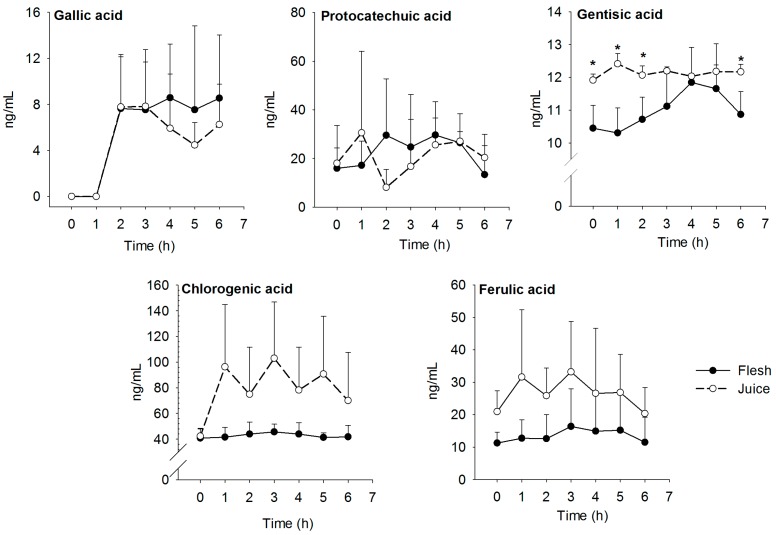
Average plasma concentration of gallic, protocatechuic, gentisic, chlorogenic, and ferulic acid in plasma (ng/mL). Blood samples were collected after consumption of ‘Ataulfo’ mango flesh (-•-) and juice (-ͦ-). * Statistically significant differences (*p* < 0.05) between mango matrices.

Five phenolic acids present in ‘Ataulfo’ mango flesh and juice were detected in plasma (gallic, chlorogenic, protocatechuic, ferulic, and gentisic acid); these results are presented in Figure 3. The pharmacokinetic parameters of the phenolic compounds are presented in Table 2. The C_max_ of chlorogenic and ferulic acid was significantly higher in subjects that consumed mango juice. The relative absorption was estimated from the AUC and dose-adjusted (AUC/dose); chlorogenic and ferulic acid showed statistically significant differences between matrices (higher values in the juice group). The five phenolic compounds detected in plasma reached C_max_ at 2–4 h after mango consumption. The T_max_ of all detected phenolic acids was statistically similar between matrices, but the T_max_ of the juice group was slightly shorter than that of the flesh. Consistent with mango juice having the highest C_max_, the AUC of chlorogenic and ferulic acid was higher in the juice group.

**Figure 4:**
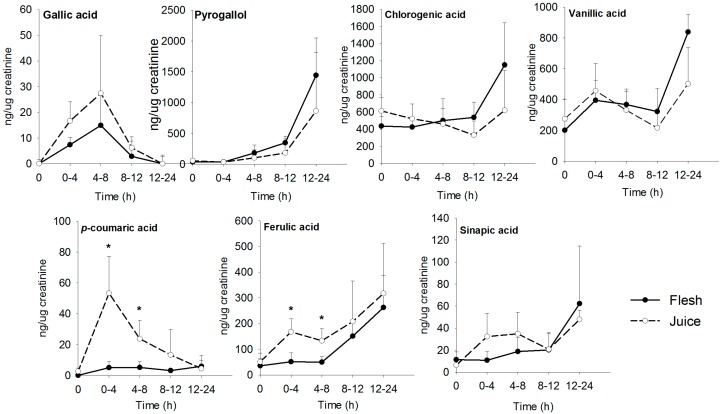
Phenolic compounds detected in urine. Samples were collected after consumption of ‘Ataulfo’ mango flesh (-•-) and juice (-ͦ-). * Statistically significant differences (*p* < 0.05) between mango matrices.

Six major phenolic acids were detected in urine, including chlorogenic, vanillic, ferulic, sinapic, gallic, and *p*-coumaric acid; results are presented in Figure 4. Pyrogallol was not detected in mango samples, but was found in urine. *p*-coumaric and ferulic acids had significantly higher excretions in the juice group at 0–4 and 4–8 h; the remaining metabolites were not significantly different between matrices. All phenolic compounds detected in urine were excreted 8–24 h after mango consumption. Interestingly, the concentration of gallic acid excreted in urine decreased with time, in parallel with an increase in pyrogallol. Pearson’s correlation coefficients (*r*) between gallic acid and pyrogallol were −0.42 for flesh and −0.47 for juice, indicating a linear negative correlation between the urinary excretion of these compounds. Higher AUC/dose excretions were found for vanillic and sinapic acids; the values were higher in the juice group.

### 3.3. Antioxidant Capacity

**Figure 5:**
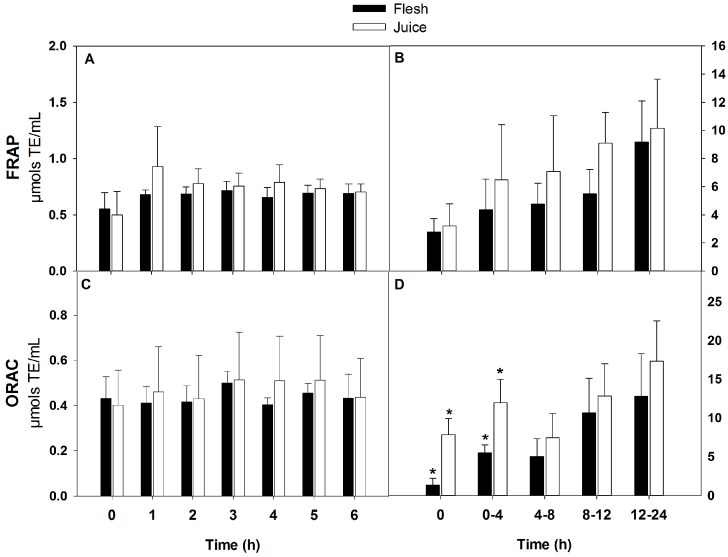
Antioxidant capacity of plasma (**A**) and urine (**B**), as determined with the ferric reducing antioxidant power (FRAP) assay. Antioxidant capacity of plasma (**C**) and urine (**D**), as determined with the oxygen radical absorbance capacity (ORAC) assay. * Statistically significant differences (*p* < 0.05) between mango matrices.

The results of plasma and urine antioxidant capacity are shown in Figure 5. No significant changes were found in antioxidant capacity values of plasma and urine at any time point. Although no significant differences were found between mango matrices, the antioxidant capacity as measured by two different assays (FRAP and ORAC) showed slightly higher plasma antioxidant capacity values in the juice group. The antioxidant capacity values of urine increased with time, possibly due to the increased excretion of phenolic acids. No significant differences were found with the FRAP assay. The ORAC assay showed significant differences between food matrices at baseline (time 0) and at the first urine sampling (0–4 h). The highest antioxidant capacity was found at 8–24 h after mango consumption. This is positively associated with the increased concentration of phenolic compounds in urine to these times (FRAP and phenolic compounds from flesh, *r* = 0.99; ORAC and phenolic compounds from flesh *r* = 0.73; FRAP and phenolic compounds from juice *r* = 0.99; ORAC and phenolic compounds from juice *r* = 0.80).

## 4. Discussion

Phenolics contribute to the inverse correlation documented between consumption of plant foods and chronic diseases, such as metabolic disorders and cancers, through mechanisms that include antioxidant, anti-inflammatory, and anti-proliferative effects [5,25]. A substantial amount of preclinical and clinical data illustrates that phenolics from plant foods are bioavailable and capable of enhancing antioxidant capacity values in humans, while also exerting multi-organ actions that promote health [26,27]. ‘Ataulfo’ mango fruit has the highest content of total phenolics among other commercial varieties of mango [2]. In this study, we investigated the bioavailability of phenolic acids of ‘Ataulfo’ in healthy adult men. We also studied whether their bioavailability would differ when they were delivered in different mango forms (flesh vs. juice), because phenolics bound tightly to flesh fiber might be less bioaccessible and bioavailable.

Our results are in agreement with reports by other authors [2,28], where phenolic acids are the major phenolic compounds in ‘Ataulfo’ mango fruit, and specifically, gallic acid and its derivatives are the most representative. Gallic acids (*m*-digallic acid and *m*-trigallic acids), gallotannins, and mangiferin are among the main phenolic compounds identified in the flesh of different mango cultivars (’Ataulfo’, ‘Alphonso’, ‘Kitchener’, ‘Abu Samaka’, ‘Keitt’) [2,28,29,30]. Our mango flesh samples contained 33.04 mg/kg of gallic acid, while Ramirez et al. [31] reported 6 mg/kg of gallic acid in ‘Pica’ mango and 17 mg/kg of gallic acid in ‘Tommy Atkins’ mango. Our ‘Ataulfo’ mango juice samples had 31.8 mg/kg of gallic acid.

Five phenolic compounds were detected in plasma after mango flesh and juice consumption, and the concentration of gentisic acid was significantly higher in the juice group. Caffeic acid was detected in the mango samples, but not in plasma or urine. This could be attributed to an oxidative degradation in the GI tract, low concentration in plasma/urine or quick absorption (<1 h) and tissue distribution, or metabolism into ferulic acid [32]. Phenolic compounds identified in plasma showed a T_max_ of 1–4 h after mango consumption, suggesting that the absorption of these phenolics occurred in the small intestine [33]. Even though some phenolic compounds such as gallic, chlorogenic, caffeic, and *p*-coumaric acids can be absorbed in the stomach via monocarboxylic acid transporters (MCTs) within 5 min after gastric administration [34], the small intestine is known to be the major site for the absorption of phenolic acids. The T_max_ of gallic acid from juice was 3.5 ± 1.0 h, which is less than the T_max_ of the same compound, as compared to its ingestion from tea (T_max_: 1.39 ± 0.21 h) and red wine (T_max_: <1.5 h) [35,36]. Both mango matrices contain similar amounts of the individual phenolic compounds, therefore the AUC/dose showed no difference, except for chlorogenic and ferulic acids.

Six phenolic compounds were detected in urine, plus one microbial metabolite, and the excretion of *p*-coumaric and ferulic acid was significantly higher in the juice group. Protocatechuic and gentisic acids were detected in plasma, but not in urine. Vanillic and sinapic acids were detected in urine but not in plasma. Excretion rates were maximum at 8–24 h. Free and polymeric gallic acid is the main phenolic acid in ‘Ataulfo’ mango. This phenolic acid is absorbed in the small intestine, but a percentage of this compound is not bioaccessible in the small intestine and reaches the colon, where it is apparently decarboxylated into pyrogallol by microbial gallic acid decarboxylase [37,38,39]. Pyrogallol has been previously reported as a metabolite after consumption of ‘Keitt’ mango, berry fruits, Concord grape juice, and black tea [37,39,40]. Stalmach, Edwards, Wightman, and Crozier [40] reported urinary pyrogallol at later time points after ingestion of Concord grape juice, but this compound was not observed in the urine of ileostomized patients, which strongly suggests a colonic origin of this metabolite. This trend was expected, as previous studies have identified increased concentrations of gallic acid microbial metabolites in plasma and urine at 6–8 h from baseline [37,41].

As previously mentioned, the matrix of different foods may favor or hinder the bioaccessibility and bioactive responses of phenolic compounds in vivo [9]. In particular, because of the physicochemical properties of dietary fiber, it can delay the release and absorption of macronutrients, some minerals and trace elements, as well as phytochemicals, such as phenolic compounds and carotenoids [42]. Food processing such as macerating, grinding, fermentations, and/or mild heating may improve the bioaccessibility and bioavailability of phenolics, most likely as a result of disruption of the cell walls (dietary fiber) of plant tissues, dissociation of bioactive compound-matrix complexes, or transformation into more active molecular structures [9]. In our study, mango flesh represents the raw food, without any modifications to the matrix, while mango juice is the soluble part that results from the removal of the fiber. Our data showed that processing mango flesh into juice can increase the Cmax of some phenolic acids, while favoring the excretion of others. This could be attributed to complex carbohydrates present in the mango matrix, which are mainly non-starch, of which pectin is the predominant compound, but hemicellulose and arabinogalactans may also be present [43,44,45]. Other authors have also reported important interactions between phenolic compounds and macromolecules, such as proteins. For example, Quintero-Flórez et al. [46] show that some of the phenolic compounds used in our study (*p*-coumaric acid, caffeic acid, vanillic acid, protocatechuic acid) and specific proteins found in olive oil (mucin) rapidly interact (1 min) to form insoluble precipitates, which suggests that the bioavailability of both phenolics and proteins may change as a consequence of these interactions.

Normal postprandial metabolic and oxidative processes produce reactive oxygen species with oxidative effects, which reduce the plasma antioxidant capacity [47]. This pattern could be related to the plasma antioxidant capacity values found in this study. Some of the absorbed antioxidants (i.e., phenolic compounds or vitamins) could exert their antioxidant power in plasma, and as a result, they can partially attenuate the oxidative stress, maintaining or increasing plasma antioxidant capacity. The higher antioxidant capacity in urine is attributed to the higher concentration of phenolic compounds excreted, but urinary pyrogallol may have also contributed to this effect.

## 5. Conclusions

We administered ‘Ataulfo’ mango to healthy subjects in two different forms, flesh or juice. We determined that the bioavailability and antioxidant capacity of the phenolic compounds are preserved, or may increase, when the mango is processed into juice. Consumption of ‘Ataulfo’ mango has the potential to increase the concentration of phenolic acids in humans, which have been shown to exert anti-inflammatory and anti-carcinogenic effects. The consumption of mango, as flesh or juice, is highly recommended as a source of health-promoting phytochemicals. Nevertheless, further studies are still required to determine the preventive or ameliorating effects of mango bioactives against specific diseases or metabolic aggressions.

## Figures and Tables

**Table 1:** Content of phenolic compounds in ‘Ataulfo’ mango flesh and juice by HPLC-ECD analysis.

No.	Phenolic Compound	Mango Flesh	Mango Juice
		(mg/500 g of Fresh Weight)	(mg/721 g of Fresh Weight)
1	*p*-coumaric acid	19.36 ± 2.46	16.63 ± 1.24
2	gallic acid	16.52 ± 0.95	15.90 ± 0.34
3	chlorogenic acid	17.96 ± 0.50	7.32 ± 0.53
4	ferulic acid	1.94 ± 0.28	1.59 ± 0.02
5	vanillic acid	1.07 ± 0.06	0.87 ± 0.02
6	protocatechuic acid	0.41 ± 0.02	0.53 ± 0.03
7	gentisic acid	0.24 ± 0.01	0.18 ± 0.03
8	sinapic acid	0.06 ± 0.00	0.06 ± 0.00
9	caffeic acid	0.03 ± 0.00	0.03 ± 0.00
Total		47.60 ± 3.72	43.24 ± 0.28

**Table 2:** Pharmacokinetic parameters after consuming ‘Ataulfo’ mango flesh and juice.

Phenolic Compound	Flesh				Juice			
	C_max_ (ng/mL)	T_max_ (h)	AUC (ng h/mL)	AUC/Dose ((ng h/mL)/mg)	C_max_ (ng/mL)	T_max_ (h)	AUC (ng h/mL)	AUC/Dose ((ng h/mL)/mg)
Chlorogenic acid	49.7 ± 7.3 *	3.5 ± 1.4	208.7 ± 24.5 *	11.5 ± 1.78 *	109.7 ± 0.26 *	2.5 ± 1.8	366.9 ± 130.7 *	50.12 ± 15.5 *
Protocatechuic acid	30.8 ± 13.3	3.5 ± 2.0	141.4 ± 73.9	344.8 ± 138.3	34.5 ± 18.0	3.7 ± 1.7	108.6 ± 5.4	204.90 ± 9.9
Ferulic acid	16.5 ± 3.9 *	2.8 ± 2.1	60.2 ± 22.7 *	31.0 ± 9.63 *	32.7 ± 10.9 *	2.3 ± 1.5	133.4 ± 47.7 *	83.8 ± 30.2 *
Gentisic acid	11.8 ± 2.1	4.0 ± 1.4	53.1 ± 8.3	221.2 ± 34.0	12.2 ± 0.2	2.8 ± 1.9	50.9 ± 11.0	282.7 ± 59.1
Gallic acid	8.7 ± 1.7	4.4 ± 1.1	36.9 ± 24.3	2.2 ± 0.91	7.9 ± 4.7	3.5 ± 1.0	38.5 ± 14.5	2.4 ± 0.94

Values are means ± standard deviation (SD) (*n* = 12) of plasma phenolic compounds. * Statistically significant differences (*p* < 0.05) between mango matrices. C_max_: maximum concentration. T_max_: maximum time. AUC: area under the curve from time 0 to 6 h.
